# The Role of Postoperative Irradiation in the Treatment of Locally Recurrent Incompletely Resected Extra-Abdominal Desmoid Tumors

**DOI:** 10.1080/13577140410001710512

**Published:** 2004

**Authors:** James Fontanesi, Michael P. Mott, Michael J. Kraut, David P. Lucas, Peter R. Miller

**Affiliations:** 1 Department of Radiation Oncology Barbara Ann Karmanos Cancer Institute Wayne State University School of Medicine Detroit MI USA; 2 Department of Orthopaedic Surgery Barbara Ann Karmanos Cancer Institute Wayne State University School of Medicine Detroit MI USA; 3 Department of Pathology Barbara Ann Karmanos Cancer Institute Wayne State University School of Medicine Detroit MI USA; 4 Department of Radiology Barbara Ann Karmanos Cancer Institute Wayne State University School of Medicine Detroit MI USA; 5 Department of Medicine Barbara Ann Karmanos Cancer Institute Wayne State University School of Medicine Detroit MI USA

## Abstract

*Background*: To define the efficacy of postoperative irradiation in patients with recurrent extra-abdominal desmoid tumors in whom surgical intervention has resulted in microscopically or grossly positive surgical margins.

*Methods*: A retrospective analysis was performed on all patients referred to the department of radiation oncology at the Detroit Medical Center with a diagnosis of recurrent extra-abdominal desmoid tumor. This analysis includes all patients seen from 1 January 1990 through 31 December 1999. A total of 11 patients were treated to 13 sites. Ten had microscopically positive margins and three had gross residual disease. Three patients were noted to have multifocal disease at the time of initial representation. Local control, survival, follow-up, and subsequent development of new tumors are measured from the last day of treatment with irradiation.

*Results*: Thirteen sites were treated. Seven patients had received chemotherapy/hormonal therapy prior to surgery and/or irradiation. The most commonly used drug was tamoxifen (*n*=6). The type of radiation delivered included external beam irradiation alone (*n*=3), combined external beam irradiation and brachytherapy (*n*=4), brachytherapy alone (*n*=3) and 252-Cf neutron brachytherapy alone (*n*=3). Follow-up has ranged from 29 to 115 months (median=76 months). Three patients have failed locally at 17, 24 and 29 months. One of these was treated for gross residual disease. No patient has died of tumor-related causes. Salvage at the failed sites was possible in twom of three with re-irradiation using external neutrons and/or aggressive surgical intervention and systemic therapy. Complications were most often noted to include decrease range in motion, especially in joint areas, and skin reactions which were normal in presentation. In one site there was development soft tissue necrosis.

*Conclusion*: Based on our experience we recommend postoperative irradiation for all recurrent extra-abdominal desmoid lesions with microscopically or grossly positive surgical margins. Furthermore, patients with recurrent desmoid tumors involving the bony structures of the hand or feet are poor candidates for brachytherapy alone. For patients with extremity lesions, brachytherapy may be a reasonable treatment option provided adequate margins around the tumor bed are covered. The continued recommended use of irradiation in this group of patients is warranted.


*Sarcoma*, June/September 2004, *VOL. 8, NO. 2/3, 83–86*

Taylor & Francis healthsciences

ORIGINAL ARTICLE

### The role of postoperative irradiation in the treatment of locally recurrent incompletely resected extra-abdominal desmoid tumors

JAMES FONTANESI[1], MICHAEL P. MOTT[2], MICHAEL J. KRAUT[5], DAVID P. LUCAS[3] & PETER R. MILLER[4]

*Departments of Radiation [1]Oncology, [2]Orthopaedic Surgery, [3]Pathology, [4]Radiology and [5]Medicine, Barbara Ann Karmanos Cancer Institute, Wayne State University School of Medicine, Detroit, MI, USA*

**Précis**

For patients who undergo re-resection for recurrent extra abdominal desmoid tumor, and in whom microscopically or grossly positive margins are found, the use of postoperative radiation is not only warranted, but is critical in the ability to establish local control. We recommend total doses of at least 50 Gy for microscopic positive surgical margins and 56 Gy for gross residual surgical margins. We recommend the use of external beam irradiation alone for patients who have involvement in the hand and plantar regions, while in the remaining areas treatment using external beam irradiation, brachytherapy alone, or a combination of external beam with brachytherapy may be utilized.

**Abstract**

*Background*: To define the efficacy of postoperative irradiation in patients with recurrent extra-abdominal desmoid tumors in whom surgical intervention has resulted in microscopically or grossly positive surgical margins.

*Methods*: A retrospective analysis was performed on all patients referred to the department of radiation oncology at the Detroit Medical Center with a diagnosis of recurrent extra-abdominal desmoid tumor. This analysis includes all patients seen from 1 January 1990 through 31 December 1999. A total of 11 patients were treated to 13 sites. Ten had microscopically positive margins and three had gross residual disease. Three patients were noted to have multifocal disease at the time of initial representation. Local control, survival, follow-up, and subsequent development of new tumors are measured from the last day of treatment with irradiation.

*Results*: Thirteen sites were treated. Seven patients had received chemotherapy/hormonal therapy prior to surgery and/or irradiation. The most commonly used drug was tamoxifen ($n=6$). The type of radiation delivered included external beam irradiation alone ($n=3$), combined external beam irradiation and brachytherapy ($n=4$), brachytherapy alone ($n=3$) and 252-Cf neutron brachytherapy alone ($n=3$). Follow-up has ranged from 29 to 115 months (median = 76 months). Three patients have failed locally at 17, 24 and 29 months. One of these was treated for gross residual disease. No patient has died of tumor-related causes. Salvage at the failed sites was possible in twom of three with re-irradiation using external neutrons and/or aggressive surgical intervention and systemic therapy. Complications were most often noted to include decrease range in motion, especially in joint areas, and skin reactions which were normal in presentation. In one site there was development soft tissue necrosis.

*Conclusion*: Based on our experience we recommend postoperative irradiation for all recurrent extra-abdominal desmoid lesions with microscopically or grossly positive surgical margins. Furthermore, patients with recurrent desmoid tumors involving the bony structures of the hand or feet are poor candidates for brachytherapy alone. For patients with extremity lesions, brachytherapy may be a reasonable treatment option provided adequate margins around the tumor bed are covered. The continued recommended use of irradiation in this group of patients is warranted.

**Key words:** *brachytherapy, radiation, neutrons, desmoid tumor*

### Introduction

Treatment decision for desmoid tumors following surgical intervention continues to be controversial. There is no clear indication as to which patients may benefit from additional therapy following surgical removal, as no randomized trial has ever been completed. However, for patients with gross or microscopic residual disease or for those who have recurrence, regardless of margin status, the use of irradiation appears to improve local control.[1–6] There have also been recent attempts to define whether chemotherapy and/or hormonal therapy might also play a role in the treatment of desmoid tumors, although less than impressive results have been reported.[7,8]

The decision to recommend radiation must be weighed against the knowledge that this is a

1357-714X print/1369–1643 online © 2004 Taylor & Francis Ltd
DOI: 10.1080/13577140410001710512

'benign' disease, and the fact that potential second malignancies may be result of intervention.[9] While many reports have described the use of irradiation for both intra- and extra-abdominal desmoid tumors, few have directed analysis in an attempt to define the role of postoperative irradiation in patients who were treated for recurrent extra-abdominal desmoid tumors in whom resection has resulted in either gross or microscopic residual disease.

Our aim was to review the experience at our center to determine whether justification could be made for the routine use of postoperative irradiation in this specific patient population, and to determine how best to deliver the irradiation.

### Methods and materials

All patients treated for recurrent desmoid tumor and in whom re-resection resulted in either gross or microscopically positive margin were retrospectively reviewed. The review period was from 1 January 1990 through December 1999. A total of 11 patients were identified. Time from initial therapy to diagnosis of recurrence ranged from 3 to 50 months (median = 5 months). Size of relapse, measured by the greatest dimension on imaging, ranged from 3 to 23 cm. Thirteen sites were treated in these 11 patients. Time to failure and local control, complications and all the parameters evaluated relating to time are calculated from the last day of radiation treatment.

All patients had their pathological slides reviewed by one of the authors (DPL). Tumor size, surgical margins and other pathological information were recorded. Patient demographics, and treatment parameters were recorded from hospital charts, including the age of diagnosis, sex, race, site and size of lesion, dates of surgical procedures including biopsies, the type of surgical procedures performed, brachytherapy and/or external beam irradiation doses, chemotherapy and complications. Patients with multicentric disease were also identified. Pathologically, margins were considered microscopically positive if tumor cells were within 3 mm of the inked margins, and grossly positive if the margin of resection was positive.

There were 11 patients in whom 13 sites were treated. There were nine females and two males. Ages ranged from 13 to 66 years at the time of initial presentation (median $n = 27$ years). Follow-up for all patients has ranged from 29 to 115 months (median = 60 months).

The initial site of presentation was as follows below: lower extremity ($n = 6$), buttock ($n = 2$), shoulder/trunk ($n = 3$), upper extremity ($n = 2$). All patients presented with either microscopic residual disease ($n = 10$) or gross residual disease ($n = 3$). Radiation was initiated within 2 weeks of surgical intervention on all patients.

Seven patients received chemotherapy/hormonal therapy prior to re-resection and radiation. Tamoxifen was the most commonly used agent ($n = 6$). No patient had a tumor response to systemic therapy.

Six patients received brachytherapy as a sole method of irradiation. Each of these patients received twice daily treatment utilizing high dose rate remote afterloading iridium-192 ($n = 3$) or manually loaded californium-252, a neutron-emitting brachytherapy source available at the Detroit Medical Center. Four patients received combination of brachytherapy and external beam irradiation, with each brachytherapy application being delivered twice daily followed by once daily external beam irradiation using fraction sizes of 180–200 cGy. Three patients received external beam irradiation alone. These received once daily irradiation using 180–200 cGy per fraction. Total doses, including conversion of high-dose rate brachytherapy to low-dose rate brachytherapy and use of an RBE of 4.5 for the Cf-252 brachytherapy, ranged from 46 to 64 Gy. There was no identifiable departmental choice for each of the treatment regimens.

### Results

Ten of 13 sites maintained local control between 32 and 115 months (median $n = 82$ months). Each of the three patients treated with external beam irradiation alone, or in combination with brachytherapy, maintained the local control.

In those patients receiving only brachytherapy, local failure has developed in three patients at 17, 24 and 29 months. Two of the three receiving Cf-252 brachytherapy failed. The first Cf-252 patient to fail was treated for gross residual disease which was encased around the major neurovascular bundles of the upper extremity which, if resected, would have resulted in a dysfunctional limb. This patient has been salvaged with combined surgery and multi-agent chemotherapy. This patient remains NED at 12 months. The second Cf-252 patient to fail had tumor that intertwined in the bones of the foot. This patient was salvaged with amputation. The third failure was in an upper extremity lesion with microscopic margins who received IR-192 HDR brachytherapy. The dose schedule delivered 350 cGy to a 0.5-cm margin. This was delivered b.i.d. for 6 days. Re-resection followed by multi-agent chemotherapy has rendered this patient NED at 26 months. In this patient, the dose was delivered to 0.5 cm around the area marked with surgical clips by the surgeon. A twice daily dose of 350 cGy was delivered for 6 days. In addition, each of these three patients had received tamoxifen prior to surgical intervention and irradiation. There was no tumor response to this agent in any of these three patients.

No patient has died as a result of this disease.

A single patient experienced an unexpected grade 4 complication. In that patient soft tissue necrosis developed following brachytherapy and external beam irradiation. Review of the treatment plan retrospectively did not demonstrate any unusual dose in homogeneity or other factors that would account for this problem. The remainder of the patients had Grade I/Grade II skin reactions. Two patients also reported, suffered from a decrease in range of motion which improved with an 85% of normal function following physical therapy.

### Discussion

Since 1994, there have been a number of published reports that have dealt with the prognostic factors and treatments for aggressive neurofibromatosis/desmoid tumors. These series have reported on various cohorts of patients who have included both intra- and extra-abdominal desmoid lesions, primary and recurrent disease and both adult and pediatric patients.[1–6] However, there are several recurrent themes to most of these series and they include the importance of the status of the surgical margins, whether treatment is in the initial postoperative setting or is recurrent and the total dose of radiation which is used.

The use of postoperative irradiation, in recurrent setting, has reported 5-year local control rates between 75 and 81% when irradiation is delivered, while local failure rates as high as 100% were noted when postoperative irradiation is omitted.[1–3,5,10] Our presented series has a similar local control rate (76%) with a median follow-up of over 6 years. Where we differ is in the fact that all sites had surgical margins that we would consider to be close or positive using the definition used by Nuyttun in his meta analysis.

In that review, Nuyttens reviewed 22 series published between 1983 and 1998.[4] Three separate groups are identified in this review. They included: (A) patients receiving surgery alone, (B) those receiving surgery and irradiation, (C) those receiving irradiation alone. Group A and B patients were further subdivided by their surgical margin status. These were then analyzed to determine whether the treatment was based on initial presentation, recurrent disease or unknown tumor status. Local control for surgical patients with negative surgical margins was 72%, for those with positive margins it was 4%. Patients receiving both surgery and irradiation enjoyed a local control of 94% with negative surgical margins and 75% for positive surgical margins. In all cases use of irradiation +/− surgery was superior in establishing local control when compared to surgery alone.

This meta analysis confirms the previous institutional series and our presented data in which about 75% of patients who receive postoperative irradiation for positive surgical margins, as defined in the meta analysis, enjoy local relapse-free survival.

It is with this in mind that the randomized report by Pisters *et al.* must be reviewed. In this report there was no benefit to the use of brachytherapy in the treatment of low-grade sarcomas. They report detailed doses of 42–45 Gy being delivered to the tumor bed in patients who had no gross residual tumor, no violation of the tumor during surgery and no involvement of major neurovascular bundles. In addition, only those with localized completely resected superficial trunk and extremity sarcomas that resulted in limb salvage were included.

It may be possible that this specific cohort of patients (superficial low grade sarcomas that are completely resected with negative margins) does not need any further therapy and that the addition of irradiation would not be beneficial in the overall survival or local control, but could certainly enhance complication rates. This paper, however, does not deal with desmoid tumors and specifically it does not report on recurrent lesions nor does it include analysis of those with positive or close margins as defined by Nuyttens in the meta analysis. Thus while this paper is important in defining treatment options for the described patient cohort, direct correlations cannot be drawn as to the recommendations for treatment in relapsed desmoids with close/positive margins.

Our rationale for the use of brachytherapy was that it might offer an opportunity to give a boost to the primary tumor bed, and/or in selected cases, in which it was used as monotherapy, may have allowed for the advantage of a shortened overall time to deliver therapy. However, as our results demonstrated, the sole use of brachytherapy and specifically the use of Cf-252 did not benefit the patients and our data suggest an inferior rate of local control when brachytherapy was used alone.

Complication rates in this group of patients were similar to those reported in other series. These were reported to range from 4 to 30% at different time periods and also based on various doses. Nuttyens reported an overall complication rate of 22%, with the most frequent complication being fibrosis with limited range of motion, occurring in about 9% of patients. In our series only one patient had a significant event (a soft tissue necrosis), which healed with aggressive wound management. The incidence of fibrosis/range of motion reduction was three of 13 sites, including the site with the soft tissue necrosis. In addition, to date no second malignant neoplasm (SMN) has been diagnosed.

### Conclusion

For patients who present with desmoid tumors and in whom resection results in a microscopic residual margin or gross residual disease, the use of postoperative radiation is not only warranted, but critical in the ability to establish local control. We do not advocate the use of brachytherapy alone, but it may be used in combination with external beam irradiation. To date no local failures have occurred when total doses greater than 50 Gy have been utilized for microscopically positive margins and 56 Gy for gross residual disease. Complication rates in this special cohort of patients is acceptable, and to date no second malignancies have been diagnosed.

### References

1. Pritchard DJ, Nascimento AG, Petersen IA. Local control of extra-abdominal desmoid tumors. *J Bone Jt Surg Am* 1996; 78(6): 848–54 .
2. Ballo MT, Zagars GK, Pollack AP. Radiation therapy in the management of desmoid tumors. *Int J Radiat Oncol Biol Phys* 1998; 42(5): 1007–14.
3. Merchant NB, Lewis JJ, Woodruff JM, Leung DH, Brennan MF. Extremity and trunk desmoid tumors: a multifactorial analysis of outcome. *Cancer* 1999; 86(10): 2045–52.
4. Nyttens JJ, Rust PF, Thomas CR Jr, Turrisi AT 3rd. Surgery versus radiation therapy for patients with aggressive fibromatosis or desmoid tumors: a comparative review of 22 articles. *Cancer* 2000; 88(7): 1517–23.
5. Goy BW, Lee SP, Eilber F, Dorey F, Eckardt J, Fu Y-S, *et al.* The role of adjuvant radiotherapy in the treatment of respectable desmoid tumors. *Int J Radiat Oncol Biol Phys* 1997; 399(3): 659–65.
6. Merchant TE, Nguyen D, Walter AW, Pappo AS, Kun LE, Rao BN. Long-term results with radiation therapy for pediatric desmoid tumors. *Int J Radiat Oncol Biol Phys* 2000; 47(5): 1267–71 .
7. Pisters PW, Harrison LB, Leung DH, Woodruff JM, Casper ES, Brennan MF. Long term results of a prospective randomized trial of adjuvant brachytherapy in soft tissue sarcoma. *J Clin Oncol* 1996; 14(3): 859–68.
8. Lackner H, Urban C, Kerbl R, Schwinger W, Beham A. Noncytotoxic drug therapy in children with unresectable desmoid tumors. *Cancer* 1997; 80(2): 334–40.
9. Skapek SX, Hawk BJ, Hoffer FA, Dahl GV, Granowetter L, Gebhardt MC, Ferguson WS, Grier HE. Combination chemotherapy using vinblastine and methotrexate for the treatment of progressive desmoid tumor in children. *J Clin Oncol* 1998; 16: 3021–7.
10. Beswick SJ, Garrido MC, Fryer AA, Strange RC, Smith AG. Multiple basal cell carcinomas and malignant melanoma following radiotherapy for anklyosing spondylitis. *Clin Exp Dermatol* 2000; 25(5): 381–3.
11. Jelinek JA, Stelzer KJ, Conrad E, Bruckner J, Kliot M, Koh W, Laramore GE. The efficiecy of radiotherapy as postoperative treatment for desmoid tumor. *Int J Radiat Oncol Biol Phys* 2001; 50(1): 121–5.

## References

[PDF_00321] Ballo M. T., Zagars G. K., Pollack A. (1998). Radiation therapy in the management of desmoid tumors.. Int J Radiat Oncol Biol Phys.

[PDF_00356] Beswick S. J., Garrido M. C., Fryer A. A., Strange R. C., Smith A. G. (2000). Multiple basal cell carcinomas and malignant melanoma following radiotherapy for ankylosing spondylitis.. Clin Exp Dermatol.

[PDF_00333] Goy B. W., Lee S. P., Eilber F., Dorey F., Eckardt J., Fu Y. S., Juillard G. J., Selch M. T. (1997). The role of adjuvant radiotherapy in the treatment of resectable desmoid tumors.. Int J Radiat Oncol Biol Phys.

[PDF_00361] Jelinek J. A., Stelzer K. J., Conrad E., Bruckner J., Kliot M., Koh W., Laramore G. E. (2001). The efficacy of radiotherapy as postoperative treatment for desmoid tumors.. Int J Radiat Oncol Biol Phys.

[PDF_00346] Lackner H., Urban C., Kerbl R., Schwinger W., Beham A. (1997). Noncytotoxic drug therapy in children with unresectable desmoid tumors.. Cancer.

[PDF_00324] Merchant N. B., Lewis J. J., Woodruff J. M., Leung D. H., Brennan M. F. (1999). Extremity and trunk desmoid tumors: a multifactorial analysis of outcome.. Cancer.

[PDF_00337] Merchant T. E., Nguyen D., Walter A. W., Pappo A. S., Kun L. E., Rao B. N. (2000). Long-term results with radiation therapy for pediatric desmoid tumors.. Int J Radiat Oncol Biol Phys.

[PDF_00328] Nuyttens J. J., Rust P. F., Thomas C. R., Turrisi A. T. (2000). Surgery versus radiation therapy for patients with aggressive fibromatosis or desmoid tumors: A comparative review of 22 articles.. Cancer.

[PDF_00341] Pisters P. W., Harrison L. B., Leung D. H., Woodruff J. M., Casper E. S., Brennan M. F. (1996). Long-term results of a prospective randomized trial of adjuvant brachytherapy in soft tissue sarcoma.. J Clin Oncol.

[PDF_00318] Pritchard D. J., Nascimento A. G., Petersen I. A. (1996). Local control of extra-abdominal desmoid tumors.. J Bone Joint Surg Am.

[PDF_00350] Skapek S. X., Hawk B. J., Hoffer F. A., Dahl G. V., Granowetter L., Gebhardt M. C., Ferguson W. S., Grier H. E. (1998). Combination chemotherapy using vinblastine and methotrexate for the treatment of progressive desmoid tumor in children.. J Clin Oncol.

